# Mnt modulates Myc-driven lymphomagenesis

**DOI:** 10.1038/cdd.2017.131

**Published:** 2017-08-11

**Authors:** Kirsteen J Campbell, Cassandra J Vandenberg, Natasha S Anstee, Peter J Hurlin, Suzanne Cory

**Affiliations:** 1 Molecular Genetics of Cancer Division, The Walter and Eliza Hall Institute of Medical Research, 1G Royal Parade, Melbourne, VIC 3052, Australia; 2 Department of Medical Biology, The University of Melbourne, Melbourne, VIC 3010, Australia; 3 Shriner’s Hospital for Children, Portland, OR, USA

## Abstract

The transcriptional represser Mnt is a functional antagonist of the proto-oncoprotein Myc. Both Mnt and Myc utilise Max as an obligate partner for DNA binding, and Myc/Max and Mnt/Max complexes compete for occupancy at E-box DNA sequences in promoter regions. We have previously shown in transgenic mouse models that the phenotype and kinetics of onset of haemopoietic tumours varies with the level of Myc expression. We reasoned that a decrease in the level of Mnt would increase the functional level of Myc and accelerate Myc-driven tumorigenesis. We tested the impact of reduced Mnt in three models of *myc* transgenic mice and in *p53*^+/−^ mice. To our surprise, *mnt* heterozygosity actually slowed Myc-driven tumorigenesis in vavP-*MYC*10 and E*μ*-*myc* mice, suggesting that Mnt facilitates Myc-driven oncogenesis. To explore the underlying cause of the delay in tumour development, we enumerated Myc-driven cell populations in healthy young vavP-*MYC*10 and E*μ*-*myc* mice, expecting that the reduced rate of leukaemogenesis in *mnt* heterozygous mice would be reflected in a reduced number of preleukaemic cells, due to increased apoptosis or reduced proliferation or both. However, no differences were apparent. Furthermore, when *mnt*^+/+^ and *mnt*^+/−^ pre-B cells from healthy young E*μ*-*myc* mice were compared *in vitro*, no differences were seen in their sensitivity to apoptosis or in cell size or cell cycling. Moreover, the frequencies of apoptotic, senescent and proliferating cells were comparable *in vivo* in *mnt*^+/−^ and *mnt*^+/+^ E*μ*-*myc* lymphomas. Thus, although *mnt* heterozygosity clearly slowed lymphomagenesis in vavP-*MYC*10 and E*μ*-*myc* mice, the change(s) in cellular properties responsible for this effect remain to be identified.

c-Myc (hereafter Myc) is a basic helix–loop–helix–leucine zipper (bHLHZip) transcription factor that regulates a multitude of cellular processes, including cell growth, proliferation, metabolism, DNA damage response, malignant transformation and apoptosis.^7, 10, 14, 16, 20, 48, 49^ In normal cells, Myc expression is tightly regulated during the cell cycle but in many cancers control is abrogated, due to chromosomal translocation, mutation or amplification of the *myc* gene, or as a result of mutations affecting upstream regulatory pathways. Whether Myc targets specific groups of genes or serves as a global amplifier of the transcriptional programme inherent to individual cell types remains the subject of vigorous debate and experimentation.^27, 29, 34^ Irrespective, the level of Myc expression critically influences cellular outcome.^33^

Myc heterodimerises with a ubiquitous bHLHZip protein, Max, and binds the E-box CACGTG to activate gene transcription.^5^ Max also binds transcriptional repressors containing Myc-related bHLHZ domains, such as the Mxd proteins and Mnt.^10, 22, 55^ Since these repressors compete with Myc for available Max and also bind E boxes, they function as Myc antagonists. Hence their loss would be predicted to mimic Myc overexpression. Deletion of Mxd proteins in mice did not produce a phenotype resembling Myc overexpression, perhaps because expression of Mxd proteins is primarily confined to differentiated cells.^22^ In contrast, loss of Mnt, which is expressed in both proliferating and differentiating cells in all tissues tested,^24^ resulted in accelerated proliferation, susceptibility to transformation by oncogenic Ras and sensitivity to apoptosis, all characteristics of Myc overexpression.^22, 25, 35^ Surprisingly, these effects of Mnt knockdown were observed also in cells lacking Myc, leading to the suggestion that Myc functions as a Mnt antagonist, relieving Mnt-mediated repression, rather than by transcriptional activation as is widely believed.^35^ However, irrespective of whether Mnt controls Myc or Myc controls Mnt, the level of Mnt would be expected to influence the oncogenic potential of Myc.

Mnt can serve as a tumour suppressor, as tissue-specific deletion of Mnt in mice resulted in mammary adenocarcinomas and T-cell lymphomas.^8, 25, 47^ Conversely, Mnt overexpression mimicked Myc knockout, producing growth defects and embryonic lethality^25^ as well as reducing cell cycle entry and proliferation of MEFs *in vitro*.^53^ In further support of a tumour suppressor role, deletion of the region encompassing the *MNT* locus has been reported in human chronic lymphocytic leukaemia^12^ and in Sezary syndrome,^52^ a cutaneous T-cell lymphoma/leukaemia. A recent study has suggested that the dominant role of Mnt, at least in proliferating T cells, is suppression of Myc-driven apoptosis.^30^

Transgenic mice have been a powerful tool for analyzing the role of Myc in malignancy.^32^ Our E*μ*-*myc* transgenic mice,^1, 21^ which model the 8;14 translocation found in Burkitt’s B lymphomas, have provided many insights into Myc-driven lymphomagenesis. The overexpression of Myc produces a polyclonal increase in pre-B cells in young mice, accompanied by reduced differentiation to mature B cells.^28^ However, Myc overexpression also enhances susceptibility to apoptosis,^3, 17, 45^ which restrains progression of preleukaemic cells to malignancy. Consequently, anti-apoptotic mutations synergise with *myc* to promote malignant transformation^4, 13, 15, 40, 44, 51^ and the pre-B and B lymphomas that eventually arise in E*μ*-*myc* mice often harbour mutations that mutate p53 or its upstream regulators,^15^ as do Burkitt lymphomas.^18, 19, 31, 54^

More recently we developed several transgenic lines that express Myc via haemopoietic-specific regulatory elements from the *vav* gene.^42, 43^ The tumour phenotype of the vavP-*MYC* mice varied between lines, depending on the level of Myc achieved: high expression resulted in rapid-onset T-cell lymphomas whereas low expression resulted in late-onset myeloid tumours and intermediate levels produced both tumour types.

Reasoning that the level of Mnt would affect Myc activity, in this study we have investigated the impact of *mnt* heterozygosity on Myc-driven tumorigenesis, using both vavP-*MYC* and E*μ*-*myc* mouse models. We also investigated whether *mnt* heterozygosity influenced tumorigenesis in *p53*^+/−^ mice.

## Results

### *Mnt* heterozygosity confers reduced Mnt expression

**Figure 1:**
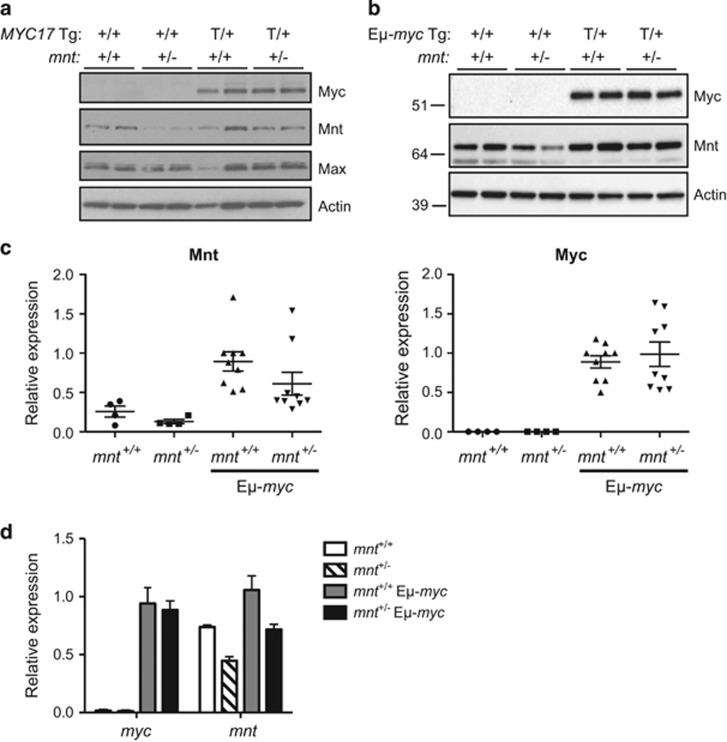
Expression analysis of *mnt*^+/+^ and *mnt*^+/−^ lymphoid cells from WT and *myc* transgenic mice. (**a**) Mnt and transgenic MYC protein expression in thymocytes from *mnt*^+/+^ and *mnt*^+/−^ WT and vavP-*MYC*17 mice. Western blots of lysates of thymocytes from four-week-old mice of the indicated genotypes, probed with antibodies specific for Myc, Mnt, Max and *β*-actin (see Materials and Methods). Each lane represents an individual mouse. Comparable results were obtained for thymocytes from *mnt*^+/+^ and *mnt*^+/−^ vavP-*MYC*10 mice. (**b**) Mnt and transgenic Myc protein expression in pre-B cells from *mnt*^+/+^ and *mnt*^+/−^ E*μ*-*myc* mice. Western blot analysis of pre-B cells (B220^+^sIg^−^) sorted by flow cytometry from bone marrow of 4-week-old mice of indicated genotypes. Each lane represents an individual mouse and MWs (kD) of markers run in parallel are indicated. Endogenous Myc was not detectable in WT control cells. (**c**) Quantification of Mnt and Myc protein expression in *mnt*^+/+^ and *mnt*^+/−^ WT and E*μ*-*myc* mice. The blot shown in (**b**) and two additional blots using independent samples were quantified using ChemiDoc and Image Lab software (Bio-Rad), with normalisation to actin expression. Expression is relative to the first *mnt*^+/+^ E*μ*-*myc* sample on each blot. (**d**) Quantitative PCR analysis of *myc* and *mnt* RNA in pre-B cells isolated using magnetic beads coated with CD19 antibody from the bone marrow of 4-week-old mice of the indicated genotypes (*n*=2–15). Data are normalised to *myc* expression in *mnt*^+/+^ E*μ*-*myc* mice

We first ascertained how *mnt* heterozygosity impacted expression of Mnt and Myc. Western blot analysis verified that the level of Mnt protein was lower in *mnt*^+/−^ compared with *mnt*^+/+^ thymocytes, both on the WT and vavP-*MYC*17 transgenic background (Figure 1a) and in pre-B cells from WT and E*μ*-*myc* mice (Figures 1b and c) and the level of transgenic Myc was unaltered. Quantitative PCR analysis confirmed that *mnt* RNA was ~2-fold lower in *mnt*^+/−^ E*μ*-*myc versus mnt*^+/+^ E*μ*-*myc* pre-B cells (Figure 1d).

### Decreased Mnt does not accelerate tumorigenesis in vavP-*MYC* transgenic mice

To study the impact of *mnt* heterozygosity on Myc-driven lymphomagenesis, we turned first to vavP-*MYC*10 mice because the level of MYC protein expressed in this line represents a critical functional threshold: When expression is increased ~2-fold (by generating mice homozygous for the transgenic locus; hereafter vavP-*MYC*10^hom^ mice), the mice develop predominantly T lymphomas rather than the mixture of T lymphomas and myeloid tumours found in mice heterozygous for the transgene.^43^ We reasoned that lowering the level of Mnt in vavP-*MYC*10^het^ mice would increase the level of MYC/Max heterodimers and drive the phenotype towards that seen in vavP-*MYC*10^hom^ mice; viz more rapid tumour onset, and primarily T lymphomas rather than a mixture of T and myeloid tumours.^43^

**Figure 2:**
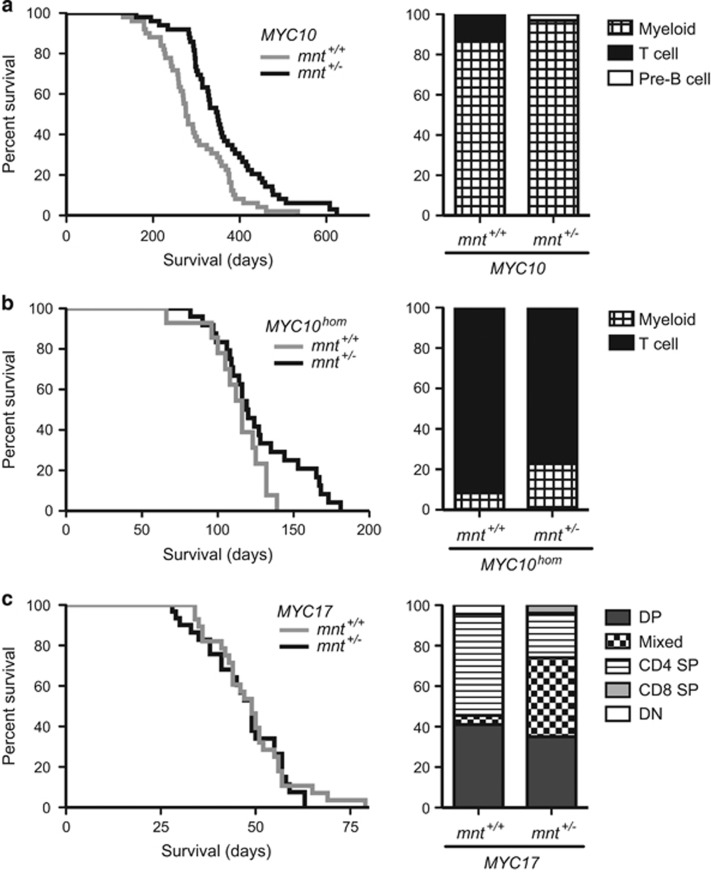
Impact of *mnt* heterozygosity on tumorigenesis in vavP-*MYC* mice. (**a**) Tumorigenesis in vavP-*MY*C10 mice. Left panel: Kaplan–Meier survival curve showing delayed morbidity in *mnt*^+/−^ vavP-*MY*C10 mice (black line, *n*=50, median survival 349 days) *versus mnt*^*+/+*^vavP-*MY*C10 mice (grey line, *n*=54, median survival 276 days); *P*<0.001, Log-rank (Mantel–Cox) test. Right panel: Incidence of different tumour types, identified by immunostaining and flow cytometry (see Materials and Methods). For *mnt*^*+/+*^vavP-*MYC*10 mice, 13% of tumours were T lymphoid (CD4^+^CD8^+^) and 87% were myeloid (Mac1^+^); for *mnt*^+/−^ vavP-*MYC10* mice, 3% of tumours were pre-B cell (CD19^+^ B220^+^ IgM^−^IgD^−^) and 97% were myeloid (Mac1^+^). (**b**) Tumorigenesis in vavP-*MYC*10^hom^ mice. Left panel: Kaplan–Meier survival curve for *mnt*^*+/+*^vavP-*MYC*10^*hom*^mice (grey line, *n*=14, median 116 days) and *mnt*^+/−^vavP-*MYC*10^*hom*^mice (black line *n*=25, median 120 days). Right panel: For *mnt*^*+/+*^vavP-*MYC*10^hom^ mice, 92% of tumours were T lymphoid (CD4^+^CD8^+^) and 8% were myeloid (Mac1^+^); for *mnt*^+/−^ vavP-*MYC*10^hom^ mice, 77% of tumours were T lymphoid (CD4^+^CD8^+^) and 23% were myeloid. (**c**) Tumorigenesis in vavP-*MYC*17 mice. Left panel: Kaplan–Meier survival curve showing similar survival of *mnt*^*+/+*^vavP-*MYC*17 mice (grey line, *n*=33; median 7 weeks) and *mnt*^+/−^ vavP-*MYC*17 mice (black line, *n*=34; median 7 weeks). Right panel: All tumours were T lymphoid. For *mnt*^*+/+*^ vavP-*MYC*17 mice, 41% were DP (CD4^+^ CD8^+^); 50% were CD4 SP (CD4^+^CD8^−^); 4.5% were DN (CD4^−^CD8^−^); 4.5% were mixed (DP and SP or DN). For *mnt*^+/−^ vavP-*MYC*17 mice, 35% were DP; 22% were CD4 SP; 4% were CD8 SP (CD4^−^CD8^+^); 39% were mixed

Cohorts of *mnt*^+/+^vavP-*MYC*10 (*n*=54) and *mnt*^+/−^vavP-*MYC*10 (*n*=50) transgenic mice) were monitored for tumour onset. Surprisingly, the outcome was the opposite from that predicted: Mnt heterozygosity (black line) resulted in *slower* tumour-associated morbidity (*P*<0.001) (Figure 2a, left panel) and, with the exception of one pre-B lymphoma, all tumours were myeloid (Figure 2a, right panel).

In *MYC*10^hom^ mice expressing higher MYC levels, *mnt* heterozygosity also retarded morbidity, although not reaching statistical significance (Figure 2b, left panel). Furthermore, the frequency of T lymphomas was reduced (77% in *mnt*^+/−^vavP-*MYC*10^hom^ mice *versus* 92% in *mnt*^+/+^vavP-*MYC*10^hom^ mice) and morbidity due to myeloid tumours increased (23% *versus* 8%) (Figure 2b, right panel). Taken together, these results suggested that Mnt heterozygosity reduces the risk of Myc-driven T lymphomagenesis in vavP-*MYC*10 transgenic mice.

Next, the impact of *mnt* heterozygosity was tested in vavP-*MYC*17 mice (Figure 2c). The level of vavP-*MYC* transgene expression is highest in this line and lymphomagenesis much faster.^43^ Therefore it was not particularly surprising to find that *mnt* heterozygosity had no impact on the kinetics of morbidity (Figure 2c, left panel). Interestingly, however, while all mice still succumbed to T lymphoma, there appeared to be a shift in tumour phenotype, a decrease in the frequency of CD4 SP (CD4^+^CD8^−^) T lymphomas (50% down to 22%) and an increase in those having a mixed (DP plus SP or DN) phenotype (4.5% up to 39%), suggestive of an impact on T lymphoid differentiation (Figure 2c, right panel).

### Decreased Mnt retards tumorigenesis in E*μ*-*myc* transgenic mice

**Figure 3:**
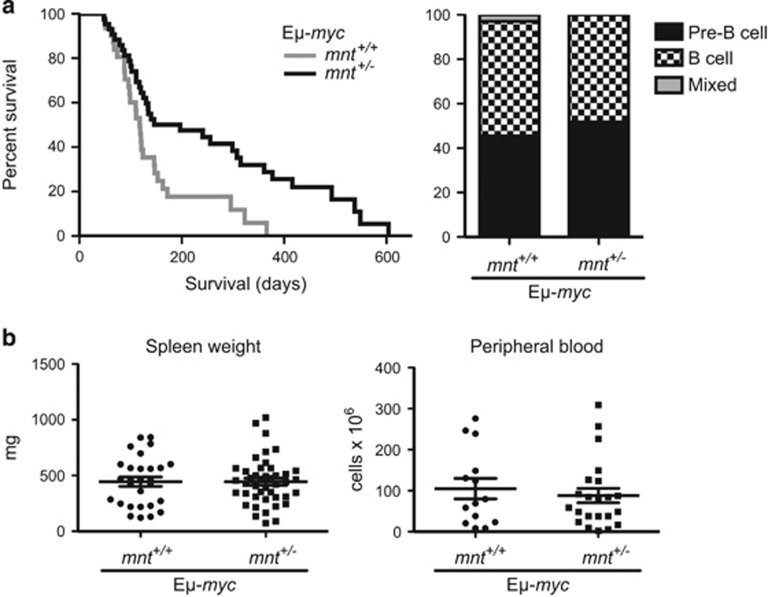
Impact of *mnt* heterozygosity on lymphomagenesis in E*μ*-*myc* mice. (**a**) Left panel: Kaplan–Meier survival curve showing delayed morbidity from tumours in *mnt*^+/−^ E*μ*-*myc* mice (black line, *n*=43, median survival 28 weeks) compared with *mnt*^*+/+*^ E*μ*-*myc* mice (grey line, *n*=31, median survival 17 weeks). *P*=0.0042. By 12 months, all *mnt*^*+/+*^ E*μ*-*myc* mice had succumbed to tumours, whereas 29% of *mnt*^+/−^ E*μ*-*myc* mice were still alive. Right panel: *mnt* heterozygosity had no impact on lymphoma phenotype in E*μ*-*myc* mice. Of 33 tumours from mnt^+/+^ E*μ*-*myc* mice analysed, 45.5% were pre-B cell, 51.5% were B cell and 3% were mixed pre-B/B-cell tumours. Similarly, of 27 tumours from *mnt*^+/−^ E*μ*-*myc* mice, 52% were pre-B cell and 48% were B-cell tumours. (**b**) Tumour burden of moribund mice. Left panel: Spleen weight of moribund *mnt*^+/+^ E*μ*-*myc* (*n*=28) and *mnt*^+/−^ E*μ*-*myc* mice (*n*=43) mice. Right panel: white blood cell count of moribund *mnt*^+/+^ E*μ*-*myc* (*n*=14) and *mnt*^+/−^ E*μ*-*myc* (*n*=22) mice. Bars represent mean ±S.E.M. No significant differences were observed

To ascertain whether *mnt* heterozygosity also impaired B lymphoid tumour development, we crossed *mnt*^+/−^ mice with E*μ*-*myc* mice, in which transgene expression is largely restricted to the B lymphoid lineage. Once again, *mnt* heterozygosity slowed rather than accelerated tumour development, the median onset being 197 days for *mnt*^+/−^ E*μ*-*myc* mice *versus* 118 days for *mnt*^+/+^ E*μ*-*myc* littermates (*P*=0.004) (Figure 3a, left panel). There was no detectable change in tumour phenotype, both genotypes developing pre-B and B-cell lymphomas at about the same relative frequency (Figure 3a, right panel). Furthermore, there was no significant difference in the severity of disease, as reflected in the spleen weight or peripheral leucocyte count of moribund mice (Figure 3b).

### Impact of Mnt heterozygosity in *p53*^+/−^ mice

**Figure 4:**
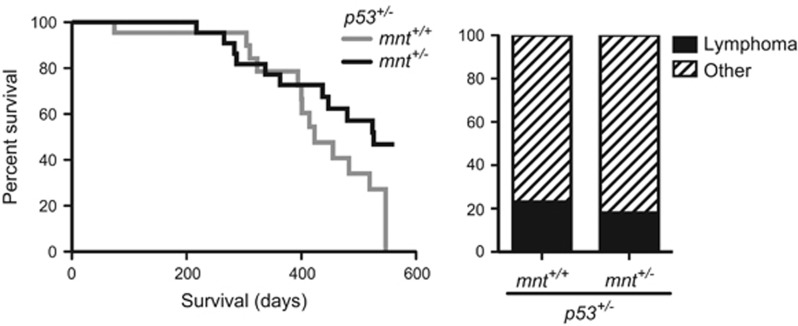
Impact of *mnt* heterozygosity in p53 heterozygous mice. Left panel: Kaplan–Meier survival curve for *mnt*^+/+^ *p53*^+/−^ mice (grey line, *n*=24, median survival 60 weeks) and *mnt* ^+/−^ *p53*^+/−^ mice (black line, *n*=25, median survival 75 weeks). Survival curves for *p53*^+/−^ *mnt*^+/+^ and *p53*^+/−^ *mnt*^+/−^ are not significantly different. Right panel: Tumour types were unaffected by *mnt* heterozygosity. Lymphoma incidence was 23% in *mnt*^+/+^ *p53*^+/−^ mice and 18% in *mnt*^+/−^ *p53*^+/−^ mice; other tumour-related deaths were predominantly due to osteosarcoma and sarcoma, as previously reported for *p53*^+/−^ mice^26^

We also tested the impact of *mnt* heterozygosity in p53-deficient mice, in which Myc can have a major impact on tumorigenesis. Lymphomas develop rapidly and at high frequency in *p53*^−/−^ mice but are rare in *p53*^+/−^ mice, which develop a range of other cancers, albeit after a long latency.^11, 26, 37^ We had reasoned that if decreased Mnt increased the level of functional Myc in lymphoid cells of p53 heterozygotes, then the frequency of lymphomas would increase and the latency shorten, as it does if an E*μ*-*myc* transgene is introduced into *p53*^+/−^ mice.^6, 23, 40^ Once again, however, *mnt* heterozygosity tended to retard tumour development, although not reaching statistical significance (Figure 4, left panel). The frequency of lymphomas *versus* other tumour types was not altered (Figure 4, right panel).

### Does Mnt heterozygosity perturb preleukaemic cell populations *in vivo*?

In contrast to earlier studies,^8, 25, 47^ these results had provided no evidence for a tumour suppressor role for Mnt in any of the mouse models tested. Indeed, they suggested instead that Mnt facilitates Myc’s oncogenic role.

**Figure 5:**
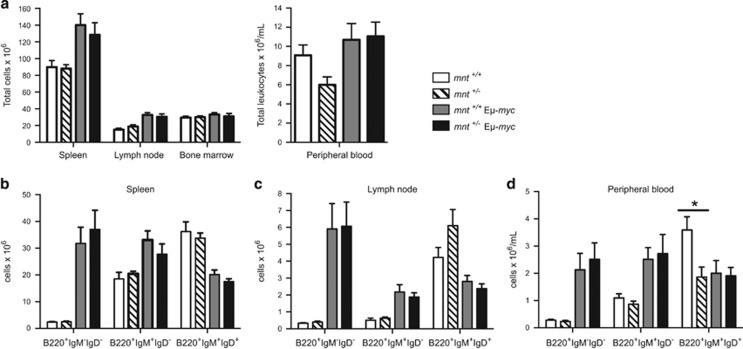
Impact of *mnt* heterozygosity on pre-tumour phenotype of E*μ*-*myc* mice. Enumeration of cell populations in 6-week-old *mnt*^+/+^ (white; *n*=5–6), *mnt*^+/−^ (diagonal stripe; *n*=6–7), *mnt*^+/+^ E*μ*-*myc* (light grey; *n*=6–7) and *mnt*^+/−^ E*μ*-*myc* (black; *n*=3–5) mice. (**a**) Total leucocytes and (**b–d**) indicated B lymphoid populations in spleen, lymph nodes, bone marrow and peripheral blood. Bars represent mean±S.E.M.; see also Supplementary Table S1. Statistical significance is shown for only *mnt*^+/+^ vs *mnt*^+/−^ and *mnt*^+/+^E*μ*-*myc* vs *mnt*^+/−^E*μ*-*myc* mice *P<0.05 as calculated by Student’s *t*-test

To gauge any impact of *mnt* heterozygosity on the premalignant Myc phenotype, we analysed haemopoietic tissues of healthy young mice, prior to tumour onset. Young E*μ*-*myc* mice harbour excessive numbers of immature B lymphoid cells, accompanied by reduced numbers of mature B cells.^28^ No major differences were apparent between 6-week-old *mnt*^+/−^ and *mnt*^*+/+*^ E*μ*-*myc* mice (Figure 5 and Supplementary Table S1). In both genotypes, the bone marrow, spleen and lymph nodes contained abnormally high numbers of pre-B (B220^+^sIg^−^) and immature B (B220^+^sIgM^+^sIgD^−^) cells, and diminished numbers of mature B cells (B220^+^sIgM^+^sIgD^+^). As expected,^28^ homoeostasis had improved by 12 weeks (Supplementary Table S2) but there were no significant differences in B lymphoid populations in *mnt*^+/−^ E*μ*-*myc* and *mnt*^+/+^ E*μ*-*myc* mice, in any tissue, at either time point.

vavP-*MYC*10 mice were analysed at 9–10 weeks of age (Supplementary Table S3). Once again, no statistically significant differences were found between *mnt*^+/+^ and *mnt*^+/−^ transgenic mice in the populations expressing the transgene, either lymphoid (B or T) or myeloid.

Due to the early onset of malignancy in the vavP-*MYC*17 line, these mice were analysed at 2 weeks. Interestingly, the transient B lymphoid hyperplasia found in these mice^42^ was significantly diminished in *mnt*^+/−^ mice compared with *mnt*^*+/+*^vavP-*MYC*17 littermates, as was the (modest) elevation in peripheral T cells (Supplementary Table S4). Thus, reduced expression of Mnt correlated with reduced Myc-driven expansion of B- and T-cell numbers in healthy young vavP-*MYC*17 mice.

In summary, analyses of preleukaemic populations in the vavP-*MYC*10 and E*μ*-*myc* mice yielded little insight into why *mnt* heterozygosity had retarded Myc-driven tumorigenesis in these mice. While changes were observed in young vavP-*MYC17* mice, these changes did not impact on the kinetics of morbidity and the unfortunate loss of this line has prevented any further analysis.

### Assessing impact of *mnt* heterozygosity on cell function

To look more closely at which Myc-induced cellular phenotypes might have been altered by reduction in Mnt, we decided to focus our attention on the E*μ*-*myc* model. Characteristics previously reported for cells overexpressing Myc include increased cell size, increased proportion of cells in the S phase of the cell cycle and increased sensitivity to apoptosis,^3, 17, 28, 41^ many of which have also been reported for cells lacking Mnt.^25, 30, 35^

**Figure 6:**
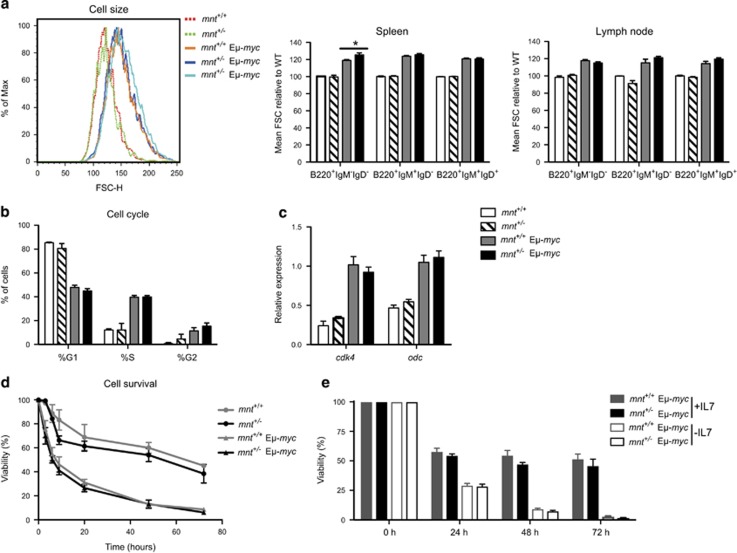
Impact of *mnt* heterozygosity on pre-neoplastic E*μ*-*myc* pre-B cells. (**a**) *Mnt* heterozygosity has no discernible impact on size of B lymphoid cells in 6-week-old *mnt*^+/+^ and *mnt*^+/−^ WT and E*μ*-*myc* mice. Cells were immunostained then gated for pre-B cells (B220^+^IgM^−^IgD^−^), immature B cells (B220^+^IgM^+^IgD^−^) and mature B cells (B220^+^IgM^+^IgD^+^) cells, and mean forward light scatter (FSC-H) determined. Values were normalised to WT cells. Left panel shows representative histogram of pre-B cells from a mnt^+/+^ (red dashed line), *mnt*^+/−^ (green dashed line), E*μ*-*myc* (orange solid line) and two *mnt*^+/−^ E*μ*-*myc* (blue solid lines) 6-week-old mice. Right panels show B lymphoid cell populations from spleen (centre) and lymph nodes (right) of 6-week-old WT (white; *n*=5–6), *mnt*^+/−^ (diagonal stripe; *n*=6–7), E*μ*-*myc* (grey; *n*=6–7) and *mnt*^+/−^ E*μ*-*myc* (black; *n*=3–5) mice. Bars represent mean±S.E.M. Statistical significance is shown for only WT vs *mnt*^+/−^ and E*μ*-*myc* vs *mnt*^+/−^ E*μ*-*myc*; *P<0.05 as calculated by Student’s *t*-test. (**b** and **c**) Mnt heterozygosity has no discernible impact on cycling of pre-B cells. (**b**) Proportion of pre-B cells in the indicated phases of the cell cycle, determined using Nicoletti stain, flow cytometry and Dean/Jett/Fox modelling using FlowJo software (see Materials and Methods). Pre-B cells were isolated from bone marrow of 4-week-old mice of the indicated genotypes using magnetic beads coated with CD19 antibody. (**c**) Quantification of *cdk4* and *odc* transcripts in sorted pre-B cells by q-PCR analysis. Expression is indicated relative to *mnt*^+/+^ E*μ*-*myc* cells. (**d** and **e**) Mnt heterozygosity has no discernible impact on apoptosis of pre-B cells *in vitro*. (**d**) Pre-malignant pre-B cells isolated from the bone marrow of 4-week-old mice using magnetic beads coated with CD19 antibody were cultured in conventional medium without additional cytokines and viability determined at the indicated time points by flow cytometry (see Materials and Methods). For each genotype, viability is expressed relative to that at *t*=0. Similar results were obtained for pre-B cells isolated using a MoFlow cell sorter (not shown). (**e**) Pre-B cells isolated from bone marrow of 4-week-old mice by flow cytometry were cultured in medium containing IL-7 as described previously.^50^ After 5 days, cells were washed three times to remove IL-7, and then replated with or without IL-7. Viability was determined at 0, 24, 48 and 72 h by flow cytometry. Results shown for *n*=6 mice of each genotype, analysed in two independent experiments; mean±S.E.M. For each genotype, viability is expressed relative to that at *t*=0

In order to avoid the complication of additional oncogenic changes, we analysed preleukaemic B lymphoid cell populations from healthy young (4- to 6-week-old) E*μ*-*myc* mice. Cell size was assessed by determining mean forward light scatter by flow cytometry (Figure 6a). Representative size profiles are shown in the left panel and the adjacent panels summarise the mean forward light scatter of pre-B (B220^+^sIg^−^), immature B (B220^+^IgM^+^IgD^−^) and mature B (B220^+^IgM^+^IgD^+^) cells, expressed relative to that of non-trangenic cells analysed contemporaneously. As expected, ^28^ the mean size of each B lymphoid cell type was greater for *myc* transgenic than non-transgenic cells. *Mnt* heterozygosity did not affect the size profile of non-transgenic or transgenic cells of any major B lymphoid type from lymph nodes (right panel) or bone marrow or peripheral blood (not shown), with the exception of pre-B cells from the spleen of *mnt*^+/−^ E*μ*-*myc* mice, which were somewhat larger than those in *mnt*^*+/+*^ E*μ*-*myc* mice (middle panel).

Cell cycle analysis of preleukaemic pre-B cells from bone marrow was performed by flow cytometry. As reported previously,^28^ the proportion of cells in the S and G2 phases was greater for cells from E*μ*-*myc* than non-transgenic mice. However, *mnt* heterozygosity had no significant impact on the cycling of either transgenic or non-transgenic pre-B cells (Figure 6b). Moreover, it did not affect the level of transcripts from the *cdk4* and *odc* genes, both of which are targeted by Myc during cell cycling (Figure 6c).^2, 20, 25^

Sorted pre-B cells were cultured in conventional medium for 3 days to ascertain their susceptibility to apoptosis (Figure 6d). As reported previously,^45^ the E*μ*-*myc* cells died at a faster rate than their non-transgenic counterparts, but *mnt* heterozygosity had only marginal impact. Similarly, when IL-7 was removed from IL-7-supported cultures of pre-B cells, *mnt* heterozygosity had no significant impact (Figure 6e).

### Impact of *mnt* heterozygosity on E*μ*-*myc* lymphoma cells

**Figure 7:**
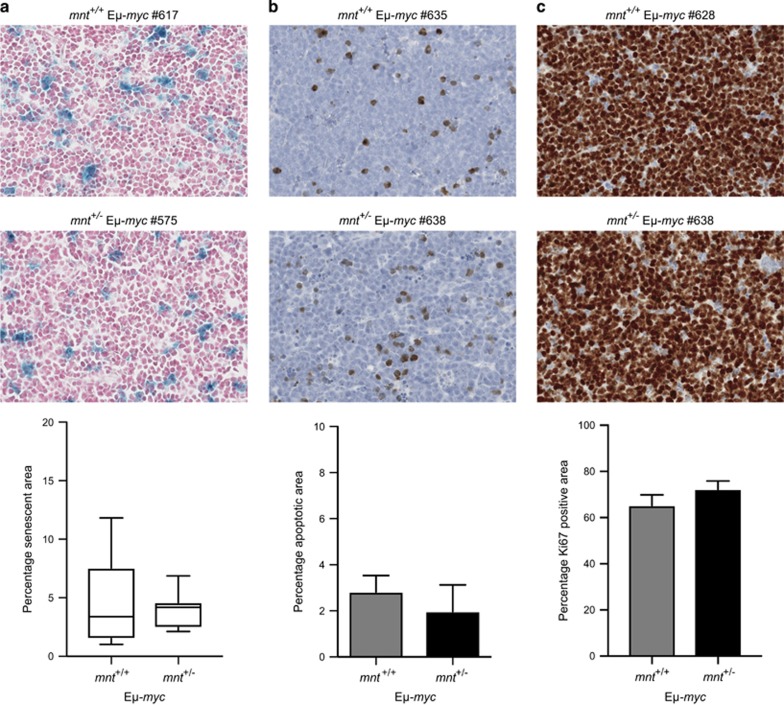
Impact of *mnt* heterozygosity on E*μ*-*myc* lymphoma cells. (**a**) *mnt* heterozygosity has no impact on senescence. Sections of lymph node lymphomas of *mnt*^+/+^ E*μ*-*myc* (*n*=12) and *mnt*^+/−^ E*μ*-*myc* (*n*=7) mice were stained for *β*-galactosidase activity, counterstained with nuclear fast red (see Materials and Methods) and scanned on an Aperio Digital Pathology Slide Scanner. *β*-Galactosidase-positive cells in extracted images were quantified using Fiji software. Data shown are mean±S.E.M. (**b**) *mnt* heterozygosity does not impact upon apoptosis. Sections of lymphomas in lymph node and/or spleen of *mnt*^+/+^ E*μ*-*myc* (*n*=10) and *mnt*^+/−^ E*μ*-*myc* mice (*n*=5) were stained using an antibody specific for cleaved caspase-3 followed by a haematoxylin counterstain (see Materials and Methods). Cells positive for cleaved caspase-3 were quantified as in (**a**) above. Data shown are mean±S.E.M. (**c**) Proliferation in E*μ*-*myc* lymphomas is not altered by *mnt* heterozygosity. Sections of lymphomas in lymph node and/or spleen of *mnt*^+/+^ E*μ*-*myc* (*n*=11) and *mnt*^+/−^ E*μ*-*myc* mice (*n*=4) were stained for Ki-67 with haematoxylin counterstain. The Ki-67 positive area was quantified as in (**a**) above. Data shown are mean±S.E.M.

Having failed to observe any changes in the preleukaemic cell populations that might account for the slower onset of tumour-associated morbidity, we decided to assess E*μ*-*myc* lymphomas to ascertain whether reduced Mnt had increased the probability of malignant Myc-driven cells undergoing cell death *in vivo* or reduced their proliferation rate. Tumour sections were stained for *β*-galactosidase activity; cleaved caspase-3 or Ki-67 and scanned digitally to quantify positive cells. No significant differences were detected between *mnt*^+/+^ and *mnt*^+/−^ E*μ*-*myc* lymphomas in the frequency of senescent (*β*-galactosidase-positive) or apoptotic (cleaved caspase-3-positive) cells (Figures 7a and b). Neither was there any difference in proliferation, as judged by quantitation of Ki-67 staining (Figure 7c).

## Discussion

The bHLHZip protein Myc activates transcription of a diverse array of genes, thereby coordinating cellular proliferation in response to cytokines, mitogens and nutrients. In normal cells, Myc is tightly regulated at transcriptional, post-transcriptional and post-translational levels, preventing inappropriate expansion of cell numbers and thereby reducing the risk of malignancy. As a further safeguard, Myc increases the propensity of cells to undergo apoptosis when cytokines and nutrients become limiting.^3, 17, 41^ Thus, somewhat paradoxically, Myc has properties of both a proto-oncoprotein and a tumour suppressor protein. The level of Myc expression can influence the balance between proliferation and apoptosis, because apoptosis was reported to be robust at high levels of Myc but minimal when Myc levels are low, perhaps reflecting differential affinities for target genes.^33^

The transcriptional repressor Mnt, also a bHLHZip protein, is a functional antagonist of Myc. Mnt competes with Myc for dimerisation with their mutual obligate partner Max and for binding at E-box sites in an overlapping set of target genes.^22^ Mnt overexpression can suppress Myc-dependent cell proliferation^24^ and tissue-specific loss of Mnt in mice resulted in mammary adenocarcinomas and T-cell lymphomas.^8, 25, 47^ Thus Mnt has been regarded as a tumour suppressor.

In this study, we set out to determine the impact of reduced Mnt levels on Myc-driven tumorigenesis. VavP-*MYC*10 mice were selected for the initial study because they are a useful barometer of MYC leukaemogenic function: Mice bearing one transgenic *MYC* allele develop both early onset T lymphomas and late myeloid tumours, and homozygosity greatly increases the frequency of T lymphomas and accelerates morbidity.^43^ We thought that reducing Mnt levels would be comparable to increasing Myc and hence expected to see accelerated tumorigenesis and increased T lymphoma frequency in mice heterozygous for vavP-*MYC*10 and heterozygous for *mnt*. In fact, the opposite occurred: *mnt*^+/−^ vavP-*MYC*10 mice developed fewer T lymphomas and morbidity was slower (Figure 2a). These results suggested that *mnt* heterozygosity had reduced susceptibility to Myc-driven T lymphomagenesis. Tumorigenesis was not significantly retarded in mice homozygous for the vavP-*MYC*10 transgene or in another T lymphoma-susceptible line (vavP-*MYC*17), possibly because Mnt heterozygosity is insufficient to counter the higher levels of Myc in these strains.

When the experiment was repeated with E*μ*-*myc* mice, in which transgene expression is largely restricted to the B lymphoid lineage, *mnt* heterozygosity again resulted in slower tumour onset. Thus, reducing Mnt by 50% can impede both B and T lymphomagenesis in two independent transgenic *myc* models.

Similar findings have been made recently using a different model: T-cell specific acute deletion of *mnt* and a ROSA-26 locus-driven *myc*^T58A^ mutant transgene.^30^ While *myc*^T58A^ mice succumbed to thymic lymphomas with a median survival of 220 days, *mnt* deletion resulted in a median survival of 355 days and none of the *mnt*^-/−^ *myc*^T58A^ mice developed thymic lymphoma. Link *et al.* also showed that Mnt-deficient thymocytes and concanavalin A-activated splenic T cells were more susceptible to apoptosis *in vitro* and concluded that the dominant physiological role of Mnt is suppression of apoptosis. The inference is that, by counteracting Myc’s capacity to promote apoptosis, Mnt facilitates Myc’s oncogenic drive.

We considered that diminished suppression of apoptosis was also a likely explanation for why *mnt* heterozygosity retards lymphomagenesis in our vavP-*MYC*10 and E*μ*-*myc* mice. Nevertheless, we were unable to provide direct evidence for this. Increased susceptibility to apoptosis might have been expected to reduce the number of Myc-driven preleukaemic cells. However, *mnt* heterozygosity provoked no diminution of pre-B-cell numbers in healthy young E*μ*-*myc* mice, nor of lymphoid (B or T) or myeloid populations in vavP-*MYC*10 mice (Supplementary Tables S1), although the transient pre-B-cell population in neonatal *mnt*^+/−^ vavP-*MYC*17 mice was significantly reduced (Supplementary Table S4). Furthermore, when we cultured preleukaemic E*μ*-*myc* pre-B cells, we found no evidence that *mnt* heterozygosity increased their susceptibility to apoptosis (Figures 6d and e).

Reasoning that *mnt* heterozygosity might be insufficient to enhance susceptibility to Myc-driven apoptosis *in vitro* but might have conferred increased susceptibility *in vivo* to particular physiological signals or to other oncogenic mutations, we quantitated cell death in sections of *mnt*^+/+^ *versus mnt*^+/−^ E*μ*-*myc* lymphomas. No significant differences were found in the frequency of either apoptotic or senescent cells (Figure 7). Furthermore, the reduced level of Mnt did not diminish the proliferation of the tumour cells, as judged by Ki-67 staining.

Although we have been unable to pinpoint the change(s) responsible for the slower onset of morbidity in our *mnt*^+/−^ *myc* transgenic mice, our study provides evidence that Mnt facilitates Myc-driven tumorigenesis.^55^ Given the plethora of genes regulated by opposing Myc/Max and Mnt/Max heterodimers, functions other than enhanced apoptosis may contribute. We are currently undertaking conditional (lymphoid-specific) homozygous deletion of *mnt* in E*μ*-*myc* and vavP-*MYC*10 mice to further explore role of Mnt in apoptosis and leukaemogenesis.

## Materials and Methods

### Mice

The vavP-*MYC* mice (which express human *MYC* cDNA^42, 43^), E*μ*-*myc* mice (which express mouse *myc* cDNA^21^) and p53-deficient mice^26^ used in this study were all on a C57BL/6 background and bred at the Walter and Eliza Hall Institute (WEHI) in accordance with the WEHI animal ethics committee regulations. The *mnt*^+/−^ mice, originally on a 129/S6 background,^46^ were backcrossed to C57BL/6 mice for several generations prior to being sent to WEHI, where backcrossing to C57BL/6 was continued. All *mnt*^+/−^ mice used in this study had been backcrossed for >9 generations. The rapid T lymphoma onset in the vavP-*MYC*17 line made it difficult to maintain a breeding colony,^43^ so cryopreserved sperm from vavP-MYC17 mice was used to fertilise oocytes from super-ovulated *mnt*^+/−^ females (67 offspring) and vavP-MYC17 *mnt*^+/−^ sperm was then collected and used to fertilise oocytes from super-ovulated wild-type females (132 offspring). Moribund mice were autopsied and organs collected for histology and immunophenotyping.

### Antibodies

Western blots, prepared by standard procedures, were probed using the following antibodies: c-Myc (N-262), Max (C-17) and Mnt (M-132) all from Santa Cruz Biotechnology (Dallas, TX, USA), or c-Myc (D84C12) from Cell Signaling Technology (Danvers, MA, USA) and Mnt (A303-626A) from Bethyl Laboratories (Montgomery, TX, USA) and *β*-actin (AC-74, Sigma-Aldrich, St. Louis, MO, USA). Blots in Figure 1b were imaged on a ChemiDoc Touch (Bio-Rad, Hercules, CA, USA) and analysed using Image Lab software (Bio-Rad). Monoclonal antibodies used for flow cytometry, produced and labelled with FITC, PE or APC in house, included the following: RB6-8C5, anti-Gr1; MI/70, anti-Mac1; YTA3.2.1, anti-CD4; 53.6.7.2, anti-CD8; Ter119, anti-erythroid marker; ID3, anti-CD19; RA3-6B2, anti-CD45R-B220; 5.1, anti-IgM; 11-26C, anti-IgD; 145-2C11, anti-CD3; E13.161.7, anti-Sca-1; 57, anti-CD43; T329.1, anti-Thy1; A20.1 anti-Ly5.1.

### Cell phenotype analysis

An ADVIA haematology analyser (Bayer, Leverkusen, Germany) or flow cytometer (LSR1; Becton Dickinson, Franklin Lakes, NJ, USA) was used to analyse peripheral blood following treatment with a buffer containing 0.168 M ammonium chloride to deplete red blood cells. Single-cell suspensions were prepared from bone marrow (one femur), thymus, lymph nodes (axillary, brachial, inguinal) and spleen. Viable cells were counted using a haemocytometer and trypan blue exclusion or enumerated on a CASY Counter (Scharfe Roche, Penzberg, Germany). Cell immunophenotype was determined by staining with surface marker-specific antibodies followed by flow cytometry analysis. Stained single-cell suspensions were sorted using a MoFlo (Cytomation, Fort Collins, CO, USA) high speed sorter. Cell viability was determined by flow cytometry following staining with propidium iodide and annexin V-FITC. Statistical significance was determined using the Student’s *t*-test (two-tailed, assuming equal variance).

### Cell cycle analysis

Pre-B cells were isolated from bone marrow using MACS columns and CD19 beads (Miltenyi, Bergisch Gladbach, Germany), resuspended in 0.1% sodium citrate, 0.1% Triton X and 50 *μ*g/ml propidium iodide (Nicoletti stain)^38^ and incubated on ice in the dark for 1 h prior to FACS analysis. Cell cycle profiles were characterised using FlowJo software (Tree Star, Ashland, OR, USA).

### Cell survival assays

Pre-B cells were isolated by magnetic purification using CD19 MicroBeads (Miltenyi Biotech, Bergisch Gladbach, Germany) and MS columns (Miltenyi Biotech) according to the manufacturer’s protocols. Cells were cultured at 0.2–0.5 × 10^6^ cells/ml in high-glucose Dulbecco’s modified Eagle’s medium supplemented with 10% foetal calf serum (Sigma-Aldrich), 50 *μ*M 2-mercaptoethanol (2-ME; Sigma-Aldrich, St Louis, MO, USA) and 100 *μ*M asparagine (Sigma-Aldrich) without additional cytokines. Cell viability was determined by staining with FITC-conjugated annexin V and propidium iodide followed by flow cytometry. IL-7 cytokine withdrawal assay was performed as published.^50^

### Histology

For cleaved caspase-3 or Ki-67 immunohistochemistry (IHC), spleens and lymph nodes from lymphoma-bearing mice were fixed in 10% formalin and then embedded in paraffin. Sections were stained for cleaved caspase-3 using the SignalStain Apoptosis (Cleaved Caspase-3) IHC Detection Kit (Cell Signaling Technology) according to the manufacturer’s protocol and counterstained with haematoxylin. Ki-67 IHC was performed using D3B5 Rabbit mAb (Cell Signaling Technology). To assess senescence, we followed the protocol of Post *et al.*^36^ Briefly, lymph nodes from lymphoma-bearing mice were fixed for 2 h in 4% paraformaldehyde, and then incubated for at least 6 h in 10% sucrose/Hanks’ Balanced Salt Solution (HBSS), followed by 15% sucrose/HBSS, then 20% sucrose/HBSS, before snap freezing in Tissue-Tek O.C.T. Compound (Sakura Finetek, Torrance, CA, USA). Cryosections were stained for SA-*β*-galactosidase as previously described,^9^ and counterstained with nuclear fast red. Complete slides were scanned on an Aperio Digital Pathology Slide Scanner and regions to be analysed were extracted using ImageScope software (Leica Biosystems, Wetzlar, Germany). Cleaved caspase-3 or *β*-galactosidase-positive cells were quantified using Fiji software (ImageJ, NIH, Bethesda, MD, USA).^39^

### PCR analysis

Lymphocyte populations were purified as described above. Total RNA was isolated using RNeasy mini kit (Qiagen, Hilden, Germany). In total, 0.2 *μ*g of RNA was reverse transcribed using the TaqmanRT system (Roche, Basel, Switzerland). Real-time PCR analysis was performed on cDNA using QuantiTect SYBR green PCR kit (Qiagen) on an ABI Prism 7900 (Applied Biosystems, Foster City, CA, USA). Relative fold expression was determined using the comparative threshold cycle method and *β*-actin. Primer sequences were as follows: *c-myc* sense caaatcctgtacctcgtccgattc, antisense cttcttgctcttcttcagagtcgc; *mnt* sense cagtccctgaagaggaagga, antisense ccggagcacacgatctatct; *ornithine decarboxylase* sense gaccttgtgaggagctgctgat, antisense tggcagtcaaactcgtccttag; *cdk4* sense cagtcagtggtgccagagatggag, antisense cagcgagggtttctccaccaag; *β-actin* sense tattggcaacgagcggttc, antisense ccatacccaagaaggaaggct.

### Statistical analysis

Statistical comparisons were made using a two-tailed Student’s *t*-test with Prism v7.0 software (GraphPad, San Diego, CA, USA). Data are shown as means±S.E.M. with *P*-values <0.05 considered statistically significant. Mouse survival analysis was carried out using GraphPad Prism (Version 7.0) and significance determined using log-rank (Mantel–Cox) test.
