# Supplementary material for: Mnt modulates Myc-driven lymphomagenesis
Source: Cell Death Differ. 2017 Aug 11;24(12):2117–26. doi: 10.1038/cdd.2017.131 (PMC5686348; doi:10.1038/cdd.2017.131)
Supplement: Supplementary Information [file cdd2017131x1.doc]

**Mnt modulates Myc-driven lymphomagenesis**

Kirsteen J Campbell, Cassandra J Vandenberg, Natasha S Anstee, Peter J Hurlin and Suzanne Cory

**SUPPLEMENTARY INFORMATION**

**Table S1**

B lymphoid composition of 6 wk-old *Eμ-myc* mice*.*

**WT *mnt ^+/-^* *Eμ-myc* *mnt^+/-^ Eμ-myc***

Peripheral blood

Total cellularity 9.1±2.6 6.0±2.3 10.7±4.5 11±4.2

B220^+^ IgM^-^IgD^-^ 0.28±0.08 0.23±0.12 2.1±1.6 2.5±1.7

B220^+^ IgM^+^IgD^-^ 1.1±0.37 0.86±0.35 2.5±1.1 2.7±2.0

B220^+^ IgM^+^IgD^+^ 3.6±1.2 1.9±1.0 * 2.0±1.2 1.9±0.85

Spleen

Total cellularity 90±19 88±14 140±38 130±41

B220^+^ IgM^-^IgD^-^ 2.4±0.43 2.5±0.78 32±17 37±20

B220^+^ IgM^+^IgD^-^ 19±9.2 20±7.9 33±16 28±11

B220^+^ IgM^+^IgD^+^ 36±18 34±14 20±8.7 17±3.1

Lymph Node

Total cellularity 15±4.1 19±6.2 33±7.8 31±10

B220^+^ IgM^-^IgD^-^ 0.34±0.04 0.40±0.16 5.9±4.2 6.1±4.1

B220^+^ IgM^+^IgD^-^ 0.51±0.27 0.61±0.23 2.2±1.2 1.9±0.73

B220^+^ IgM^+^IgD^+^ 4.2±1.3 6.1±2.9 2.8±1.0 2.4±0.79

Bone Marrow

Total cellularity 30±4.5 30±4.6 33±6.0 31±4.7

B220^+^ IgM-IgD^-^ 4.5±1.7 5.6±1.7 12±3.1 12±3.9

B220^+^ IgM^+^IgD^-^ 1.8±0.65 2.1±0.62 1.6±0.56 1.2±0.44

B220^+^ IgM^+^IgD^+^ 1.2±0.64 1.1±0.38 0.36±0.23 0.29±0.15

Values given are total nucleated cells x 10^6^ (mean ± SD), except for peripheral blood cells which are 10^6^/mL. Student’s T test of *mnt*^+/-^ mediated effects was performed for *mnt*^+/-^ (9 mice) compared to WT (6 mice) and *mnt*^+/-^ *Eμ-myc* (8 mice) compared to *Eμ-myc* (8 mice); * p< 0.05. Mice included both males and females.

**Table S2.**

B lymphoid composition of 12 wk-old *Eμ-myc* mice*.*

**WT *mnt ^+/-^* *Eμ-myc* *mnt^+/-^ Eμ-myc***

Peripheral blood

Total cellularity 6.5±2.4 5.3±2.5 7.4±3.6 5.9±2.2

B220^+^ IgM^-^IgD^-^ 0.25±0.12 0.19±0.13 0.57±0.21 0.49±0.12

B220^+^ IgM^+^IgD^-^ 0.92±0.33 0.87±0.62 1.2±0.71 0.99±0.67

B220^+^ IgM^+^IgD^+^ 2.8±1.4 2.1±1.3 1.5±0.62 1.20±0.65

Spleen

Total cellularity 85±7.5 76±10 86±19 71±10

B220^+^ IgM^-^IgD^-^ 2.1±0.43 1.8±0.63 9.5±4.3 8.8±4.3

B220^+^ IgM^+^IgD^-^ 13.4±5.6 15±5.8 13±6.1 12±2.6

B220^+^ IgM^+^IgD^+^ 37±16 33±14 21±10 16±2.5

Lymph Node

Total cellularity 14±4.0 13±1.9 18±9.6 14±5.1

B220^+^ IgM^-^IgD^-^ 0.27±0.09 0.23±0.08 1.69±2.20 0.99±0.86

B220^+^ IgM^+^IgD^-^ 0.42±0.17 0.36±0.12 0.61±0.46 0.37±0.12

B220^+^ IgM^+^IgD^+^ 4.8±1.6 4.3±0.76 2.0±1.1 1.3±0.50

Bone Marrow

Total cellularity 27±4.2 29±3.9 34±6.1 34±4.6

B220^+^ IgM^-^IgD^-^ 3.1±1.1 3.9±1.6 12±2.8 11±3.6

B220^+^ IgM^+^IgD^-^ 1.0±0.45 1.49±0.55 0.97±0.21 0.86±0.36

B220^+^ IgM^+^IgD^+^ 1.5±0.68 1.83±0.94 0.51±0.24 0.46±0.23

Values given are total nucleated cells x 10^6^ (mean ± SD), except for peripheral blood cells which are 10^6^/mL. Student’s T test of *mnt*^+/-^ mediated effects was performed for *mnt*^+/-^ (8 mice) compared to WT (7 mice) and *mnt*^+/-^ *Eμ-myc* (6 mice) compared to *Eμ-myc* (5 mice) but there were no differences having p< 0.05. Mice included both males and females.

**Table S3**

Haemopoietic composition of 9-10 week old vavP-*MYC*10 mice

**WT *mnt ^+/-^* *MYC*10 *mnt^+/-^MYC*10**

Peripheral blood

Total 9.9±3.8 9.3±3.3 8.4±3.5 5.3±1.2

CD4^+^CD8^-^ 2.1±0.93 1.5±0.76 1.4±1.0 0.60±0.15

CD4^-^CD8^+^ 1.3±0.54 0.84±0.50 0.73±0.52 0.41±0.15

B220^+^ IgM^-^IgD^-^ 0.30±0.19 0.27±0.20 0.50±0.30 0.11±0.08*

B220^+^ IgM^+^gD^+^ 4.6±1.9 3.6±0.53 4.6±2.5 2.7±0.83

Mac1^+^  0.62±0.43 0.64±0.46 0.80±0.44 0.30±0.14

Mac1^+^ Gr1^+^ 1.6±1.4 0.78±0.59 0.41±0.34 0.29±0.07

Spleen

Total 54±28 53±24 63±38 69±28

CD4^+^CD8^-^ 7.2±3.6 7.0±5.5 13±15 8.5±3.6

CD4^-^CD8^+^ 2.0±1.2 1.9±0.43 1.8±1.2 2.4±0.88

B220^+^ IgM^-^IgD^-^ 1.6±1.5 1.6±0.80 2.9±1.9 2.2±0.81

B220+ IgM^+^IgD^+^ 15±9.7 14±10 16±14 18 ±5.8

Mac1^+^ 3.9±2.6 5.5±4.2 4.4±3.4 3.8±2.0

Mac1^+^Gr1^+^ 3.2±2.7 2.0±1.6 1.6±1.4 1.7±1.1

Ter-119^+^ 16±12 7.4±4.3 18±15 15±18

LN

Total 9.0±3.1 7.4±4.6 7.9±9.0 5.7±1.8

CD4^+^CD8^-^ 3.6±1.2 2.4±1.6 2.7±3.3 2.0±0.54

CD4^-^CD8^+^ 2.2±.0.95 1.4±0.66 1.7±2.3 1.0±0.18

B220^+^ IgM^-^IgD^-^ 0.31±0.16 0.18±0.14 0.39±0.41 0.28±0.21

B220^+^ IgM^+^IgD^+^ 2.6±1.1 3.0±2.9 2.5±2.9 1.9±1.3

BM

Total 33±3.3 31±18 17±11 32±6.0*

Ter-119^+^ 8.4±5.5 11±15 7.2±5.8 6.1±6.5

Thy1^+^ 8.5±5.51 2.1±1.8* 1.6±1.8 5.2±3.1*

B220^+^ IgM^-^IgD^-^ 3.5±1.0 2.6±1.2 2.3±1.2 4.9±1.6**

B220^+^ IgM^+^IgD^+^ 2.2±0.8 1.6±0.74 0.92±0.79 1.9±0.30*

Mac1^+^ 1.0±0.50 0.85±0.51 0.40±0.28 1.2±0.93

Mac1^+^Gr1^+^ 9.6±3.6 6.0±1.6 4.4±5.6 7.6±4.1

Thymus

Total 53±33 66±51 57±31 89±36

CD4^-^CD8^-^ 4.6±3.9 4.2±2.7 3.1±2.3 4.1±2.9

CD4^+^CD8^+^ 45±30 57±46 40±19 76±32*

CD4^+^CD8^-^ 2.2±1.1 3.6±2.5 3.2±1.9 7.6±4.2*

CD4^-^CD8^+^ 2.0±1.9 1.3±0.43 1.4±0.62 1.7±0.80

Values given are total nucleated cells x 10^6^ (mean ± SD), except for peripheral blood cells which are 10^6^/mL. Student’s T test of *mnt*^+/-^ mediated effects was performed for *mnt*^+/-^ (6 mice) compared to WT (4 mice) and for *mnt*^+/-^ *MYC*10 (5 mice) compared to *MYC*10 (7 mice) * p< 0.05 ** p< 0.01. All mice were male.

**Table S4**

Haemopoietic composition of 2 wk-old vavP-*MYC*17 mice

**WT *mnt ^+/-^* *MYC*17 *mnt^+/^ MYC*17**

Peripheral blood

Total 13±2.9 11±2.1 74±17 46±25*

CD4^+^CD8^-^ 2.4±0.67 1.9±0.42 3.6±1.89 4.0±5.8

CD4^-^CD8^+^ 1.2±0.24 1.1±0.27 1.9±2.2 1.5±1.4

B220^+^ IgM^-^IgD^-^ 1.8±1.1 1.9±1.2 14±5.7 8.0±5.2

B220^+^ IgM^+^IgD^+^ 4.9±1.5 4.2±1.2 46±16 16±9.1**

Mac1^+^  1.9±1.2 0.87±0.40 7.0±2.7 4.5±3.0

Mac1^+^Gr1^+^ 0.51±0.38 0.76±0.21 2.2±0.97 1.7±0.83

Spleen

Total 66±17 67±26 190±66 120±42

CD4^+^CD8^-^ 3.6±2.4 3.5±1.8 5.6±3.7 3.4±1.5

CD4^-^CD8^+^ 1.6±0.77 1.9±1.2 2.3±2.2 1.5±0.64

B220^+^ IgM^-^IgD^-^ 7.8±4.7 6.5±5.3 21±11 17±9.7

B220+ IgM^+^IgD^+^ 23±11 21±10 35±21 20±6.9

Mac1^+^ 1.9±1.6 2.4±1.7 11±9.2 5.6±3.9

Mac1^+^Gr1^+^ 0.95±0.45 1.0±0.86 2.3±1.7 1.3±1.1

Ter-119^+^ 30±10 32 ±12 140±49 66 ±37*

LN

Total 4.7±2.4 2.8±1.6 19±14 5.1±2.3*

CD4^+^CD8^-^ 1.9±0.94 1.2±0.81 8.3±8.1 1.6±1.3

CD4^-^CD8^+^ 0.67±.0.58 0.69±0.37 2.2±1.8 0.71±0.44

B220^+^ IgM^-^IgD^-^ 0.42±0.41 0.18±0.19 2.5±1.7 0.74±0.44*

B220^+^ IgM^+^IgD^+^ 1.0±0.85 0.59±0.25 3.4±3.22 0.94±0.66

BM

Total 18 ±4.7 27±6.9* 20±5.7 20±8.6

Ter-119^+^ 8.0±1.5 13±3.3* 4.7±1.5 2.9±2.1

Thy1^+^ 0.52±0.41 0.51±0.35 0.58±0.33 1.1±1.2

B220^+^ IgM^-^IgD^-^ 5.6±1.9 9.0±3.4 6.7±3.1 9.0±3.7

B220^+^ IgM^+^IgD^+^ 1.6±0.80 2.4±1.3 1.9±0.87 1.5±0.99

Mac1^+^ 1.7±0.90 2.4±0.92 4.5±2.0 3.2±1.3

Mac1^+^Gr1^+^ 2.5±0.62 3.6±0.70* 2.3±0.63 1.9±0.40

Thymus

Total 120±28 160±36 150±28 140±59

CD4^-^CD8^-^ 4.6±2.2 4.9±1.3 6.0±2.3 5.4±3.1

CD4^+^CD8^+^ 110±22 140±36 140±28 120±45

CD4^+^CD8^-^ 9.7±4.9 14 ±3.1 7.0±5.8 11±8.9

CD4^-^CD8^+^ 3.4±1.5 3.4±1.5 3.7±2.4 5.7±4.5

Values given are total nucleated cells x 10^6^ (mean ± SD), except for peripheral blood cells which are 10^6^/mL. Student’s T test of *mnt*^+/-^ mediated effects was performed for *mnt*^+/-^ (7 mice) compared to WT (7 mice) and for *MYC*17*mnt*^+/-^ (6 mice) compared to *MYC*17 (6 mice); * p< 0.05, ** p< 0.01). Mice included both males and females.
